# The influence of information generation on destination memory

**DOI:** 10.1177/17470218231205373

**Published:** 2023-10-31

**Authors:** Raquel Pinto, Pedro B Albuquerque

**Affiliations:** School of Psychology, University of Minho, Braga, Portugal

**Keywords:** Destination memory, internal attentional focus, generation effect, personal facts, familiar proverbs

## Abstract

To remember to whom we transmit information, we rely on destination memory, with worse performance occurring when participants share personal facts (e.g., my age is . . .) compared with interesting ones (e.g., a shrimp’s heart is in its head). When reporting personal information, the internal attentional focus decreases the attentional resources available to associate that information with recipients, resulting in worse destination memory. Given that the poorer destination memory when participants transmitted personal facts was always compared with the transmission of interesting facts, in Experiment 1 (between-participants design: 41 participants) and Experiment 2 (within-participants design: 30 participants), we compared the generation and transmission of personal facts with the transmission of familiar proverbs. Again, the generation and transmission of personal facts hampered destination memory. Besides the type of information (personal vs. familiar proverbs), the conditions differed regarding the type of process (generation vs. transmission of information). To clarify the influence of generation on destination memory, in Experiment 3 (*N* = 31), participants (1) transmitted and (2) generated and transmitted familiar proverbs, and significant differences in destination memory between the conditions was not observed. In general, our experiments seem to support the assumption that transmitting personal information leads to worse destination memory not because participants generated the information but because personal facts drive the attentional focus to the self.

In our daily lives, we have multiple interpersonal interactions with different people. These social interactions put significant demands on memory because it is crucial to remember what is said and to whom we say it. We rely on destination memory to recall the person we transmitted something (Gopie & MacLeod, 2009). Research suggests that better destination memory is associated with greater communication effectiveness and better interaction with others (Gopie & MacLeod, 2009; T. L. Johnson & Jefferson, 2018) because remembering the destination of information can help avoid the social embarrassment of recounting the same story or joke to the same person repeatedly. It is also essential in the workplace, for example, when supervisors need to remember to whom they delegated a specific task.

Destination memory has been compared with source memory (i.e., our ability to remember who told us something—M. K. Johnson et al., 1993), and the results of several studies showed that destination memory was less accurate than source memory (Fischer et al., 2015; Gopie & MacLeod, 2009; Lindner et al., 2015); it is more challenging to recognise whom we shared a piece of information with than it is to recognise who told us something. According to Gopie and MacLeod (2009), when a person receives information from someone else, the incoming information and the transmitter are closely linked, making it easier to recognise the person who told us something. In other words, when people direct their attention to the incoming information, the attentional focus is also directed to the person who is providing it, leading to better source memory performance. Attention to the information and the person will benefit their association. In contrast, when a person is transmitting information to someone else, the focus is on the person sharing and on the information. This results in an internal attentional focus (i.e., higher self-focus), which leaves fewer attentional resources available to associate the outgoing information with the person to whom one is telling it. Therefore, more destination memory errors occur because fewer attentional resources are available to associate the two independent pieces of information: what was said and to whom.

The standard paradigm to study destination memory consists of two phases: the study phase and the test phase. In the study phase, participants are instructed to tell facts (e.g., *the United States Postal Service handles 40% of the world’s mail volume*) to celebrity faces (e.g., Barack Obama). Celebrity faces are often used because, in everyday life, we mainly transmit information to persons who are known or familiar to us (i.e., family, friends, and colleagues). Subsequently, participants completed two recognition memory tests in the test phase: an item memory test (i.e., memory for the facts and the faces previously presented) and a destination memory test (i.e., memory for whom a fact was transmitted). In the item memory test, a single stimulus is presented at a time (i.e., a fact or a face), half studied and half not studied (i.e., new stimuli/distractor). In the destination memory test, fact-face pairs are presented, and participants decide whether they said that fact to that face. Half of the pairs (fact-face) are shown precisely as they had been learned in the study phase, whereas the other half are re-matched.

Based on the premise that destination memory errors are committed because fewer attentional resources are available to associate independent pieces of information (e.g., facts to faces), it should be possible to impair or improve destination memory by manipulating factors that influence how people focus their attention (Barros et al., 2021; Gopie & MacLeod, 2009). For example, to increase participants’ attention focus on the destination of information, Gopie and MacLeod asked participants to say the destination person’s name before sharing the information, which enhanced destination memory performance. The authors proposed that when the name is spoken, there is an increase in the attentional focus on the context (i.e., the destination person’s face), promoting the association between the face and the fact, thus improving destination memory. Better destination memory performance also was observed when a distinctive feature was added to the destination face (Barros et al., 2021). It seems that distinctive or unique features, such as a scar or a tattoo, increase the attentional focus on the destination person, strengthening the face–fact association and consequently improving destination memory.

Besides recipient-related variables (e.g., facial distinctiveness), several studies have observed the effect of information-related variables on destination memory (e.g., the type of information transmitted). For example, in Barros et al. (2021) and several other studies, instead of general knowledge facts, the authors used proverbs, notably in studies with populations with known disorders (e.g., Alzheimer’s disease: El Haj et al., 2013; Schizophrenia: El Haj et al., 2017; Korsakoff’s syndrome: El Haj, Kessels, et al., 2016; Huntington’s disease: El Haj, Caillaud, et al., 2016) and in older samples (El Haj et al., 2013; Gopie et al., 2010), given the cognitive decline associated with these samples. The use of proverbs was justified with the assumption that when the information is more familiar to the participants, more cognitive resources are available to memorise the associations between facts and faces (El Haj et al., 2015). Indeed, a study comparing the transmission of familiar proverbs with unfamiliar proverbs in younger and older adults observed better destination memory for familiar ones (El Haj et al., 2015). When the authors presented unfamiliar proverbs, their novelty led to attention being directed towards processing the proverbs themselves, leaving fewer attentional resources to process contextual features (e.g., the face of the celebrity), which resulted in worse destination memory performance (El Haj et al., 2015). In contrast, familiar stimuli leave more attentional resources available for processing contextual information. The authors (El Haj et al., 2015) concluded that older adults showed poorer destination memory than younger adults. Moreover, better destination memory was observed for familiar faces and proverbs than for unfamiliar faces and proverbs.

Apart from general and interesting facts and familiar proverbs, the influence on destination memory of transmitting personal facts has also been explored. Gopie and MacLeod (2009, Exp. 2) asked participants to convey personal facts (e.g., *my favourite colour is* . . .) versus interesting facts (e.g., *a shrimp’s heart is in its head)*. Destination memory was worse for personal facts. The idea is that transmitting personal facts increases participants’ self-focus (i.e., the attentional focus on themselves). This internal attentional focus results in fewer attentional resources available to associate facts and faces. This result was later replicated by T. L. Johnson and Jefferson (2018).

The negative effect of self-focus was also observed for source memory. For example, in a study by M. K. Johnson et al. (1996), when people were focused on their own emotions, their memory for identifying which of two speakers told them a statement was worse compared with when they were focused on how the speaker felt (M. K. Johnson et al., 1996). Also, Jurica and Shimamura (1999) observed that when participants generated the answers to personal questions (e.g., “What is your primary means of transportation?”) or read statements (“My primary means of transportation is my bike”) presented by one of three faces, remembering the source of information was disrupted if participants were required to answer questions on person-oriented topics (e.g., individual habits, tastes, likes, and dislikes). The authors also observed increased item memory on the personal questions condition (Jurica & Shimamura, 1999). Generating answers to personal questions increased item memory more than only read statements, which can be explained by the generation effect, where self-generated material is better remembered than information that is only read, a result widely replicated (see Bertsch et al., 2007, for a review).

So, in previous studies (Gopie & MacLeod, 2009; T. L. Johnson & Jefferson, 2018), it was observed that destination memory was worse when participants generated and transmitted personal facts compared with when participants were asked to share interesting facts. It is important to note that despite the authors explaining the worse destination memory when personal facts were transmitted based on an internal attentional focus (Gopie & MacLeod, 2009; T. L. Johnson & Jefferson, 2018), the attentional focus was never directly tested. Therefore, maybe another explanation can clarify these results. For example, in addition to the type of material (personal facts vs. interesting facts), there was another difference between the two conditions: *solely transmission* when interesting facts were the information to share vs. *generation and transmission* when personal facts were conveyed. So, the worse destination memory could be attributed either to the facts being personal, to them being generated, or both.

With this set of experiments, we sought to disentangle the influence of the generation effect on destination memory to observe whether the worse destination memory was due to the transmission of personal facts or whether the information generation also affected performance. Experiment 1 compared personal fact generation and transmission with the transmission of familiar proverbs. As the worse destination memory when participants transmitted personal facts was always compared with the transmission of interesting facts, in Experiment 1, we wanted to observe whether the same pattern of results would be obtained when using familiar proverbs (which has never been done before). Also, we want to observe if the generation effect leads to better item memory, as observed in the study developed by Jurica and Shimamura (1999). Experiment 2 replicated Experiment 1, applying a different design: a within-participants design. Because most of the studies have used a between-participants design and to better understand the item memory performance, with Experiment 2, we aimed to observe whether the same pattern of results would be obtained when applying a different experimental design. In Experiment 3, we compared the generation and transmission of familiar proverbs with the transmission without the generation of familiar proverbs to observe the effect of generating non-personal information on destination memory. Information generation (i.e., familiar proverbs) was the only difference between the conditions. Thus, this set of three experiments was performed to provide robustness to the previous results (Gopie & MacLeod, 2009; T. L. Johnson & Jefferson, 2018) and to observe the possible influence of the generation effect on destination memory.

## Experiment 1

Given that the poorer destination memory when participants transmitted personal facts was always compared with the transmission of interesting facts, in Experiment 1, we wanted to observe whether the same pattern of results would be obtained when using familiar proverbs—a type of stimulus widely used in destination memory research. No studies have investigated the effect of transmitting personal facts compared with another set of facts. Maybe worse destination memory for personal facts occurs because interesting facts were used in the other condition. So perhaps the difference between conditions was not because the transmission of personal facts leads to poorer destination memory but because transmitting interesting facts attracts greater attention and leads to better destination memory.

We replicated the study of Gopie and MacLeod (2009), where the authors compared the transmission of personal facts with the transmission of interesting facts in a between-participants design. The only difference in this experiment was the material utilised in the control condition: familiar proverbs instead of interesting facts. Using familiar proverbs instead of other types of facts, like general knowledge information, allowed us to present known information in both conditions. However, despite personal facts being familiar information for the participants, this type of fact directs the attentional focus on the self (i.e., a higher internal attentional focus).

We thus expected to replicate the previously observed results (Gopie & MacLeod, 2009; T. L. Johnson & Jefferson, 2018)—worse destination memory for personal facts transmission. With this experiment, we may conclude that worse destination memory when participants transmitted personal facts is observed even when presented with another type of information in the control condition, namely, familiar proverbs.

## Method

### Participants

The sample consisted of 41 undergraduate students (37 females) aged 18 to 26 years (*M_age_* = 20, *SD* = 1.30). The sample size was calculated *a priori* through G*Power (Faul et al., 2007), targeting an independent samples t-test and using an alpha (α) of .05, a large effect size (Cohen’s *d* = .80), and statistical power of .80. The effect size was chosen considering the literature on destination memory, more specifically in Gopie and MacLeod’s (2009, Experiment 2), where the authors compared the transmission of personal facts with the transmission of interesting facts. Participants were native Portuguese speakers and had normal or corrected-to-normal vision. Written consent was obtained from all participants who received course credits for their participation. The local Ethics Committee approved this experiment.

### Materials

#### Facts

##### Familiar proverbs

Sixty Portuguese proverbs with 4 to 10 words (e.g., *“A pressa é inimiga da perfeição”*^1^) were selected from a previous study (Barros et al., 2021). Proverbs were chosen to have an average familiarity above 4 on a 5-point Likert-type scale (*M* = 4.65, *SD* = .27) that varied from 1 to 5 (1 = *very unfamiliar proverb*; 2 = *unfamiliar proverb*; 3 = *neutral proverb*; 4 = *familiar proverb*; 5 = *very familiar proverb*). In addition, to ensure that proverbs’ emotionality did not influence the results, we selected proverbs with neutral emotional valence (*M* = 3.12, *SD* = .44), with values between 2.75 and 3.80 on a Likert-type scale that ranged from 1 to 5 (1 = *very negative proverb*; 2 = *negative proverb*; 3 = *neutral proverb*; 4 = *positive proverb*; 5 = *very positive proverb*). The proverbs were presented in 14-point lowercase white font against a black background.

##### Personal facts

Sixty personal facts with 4 to 12 words (e.g., “*A minha cor favorita é . . .*”^2^) were translated and adapted to European Portuguese from the study of Gopie and MacLeod (2009), each with a blank at the end for participants to complete with their answer. The personal facts were presented in 14-point lowercase white font against a black background.

#### Faces

Sixty celebrity pictures (e.g., Barack Obama) were selected from a celebrity database validated for the Portuguese population using the same age group—young adults (Lima et al., 2021). The 60 celebrity pictures selected had over 80% recognition (*M* = 97.17; *SD* = 3.61) and 75% naming accuracy (*M* = 91.69; *SD* = 5.34). The images selected were also controlled regarding background (i.e., 30 Portuguese and 30 international) and gender (i.e., 30 male and 30 female), with all these variables being presented proportionally. All images were shown in colour against a black background with a 9 × 9 cm dimension.

### Design

The independent variables manipulated were the Type of Facts (personal facts vs. familiar proverbs) and the Type of Test Stimuli (facts vs. faces). Like Gopie and MacLeod (2009), the first variable was manipulated in a between-participants design. Half of the participants transmitted personal facts, and the other half transmitted familiar proverbs to celebrity faces. The Type of Test Stimuli was manipulated through a within-participants design. To measure our dependent variable, recognition, sensitivity (*dʹ*) score (z[P(hits)] ˗ z[P(false alarms)]) and response bias (C) score (˗[z(Hits) + z(False Alarms)]/2) were calculated for both memory tests (item memory and destination memory).^3^ Hits refer to “yes” responses to the face–fact pairs, facts, and faces that were presented in the study phase (correct “yes” answer). False alarms refer to “yes” responses to face–fact pairs, facts, and faces that were not presented in the study phase (incorrect “yes” answers). When the number of hits and/or the number of false alarms is 0 or 1, the *z* (Hits) and the *z* (False Alarms) are equal to infinite. To avoid data loss, adjustments to abstain from infinite values are in common use (Hautus, 1995; Miller, 1996). The adjustment occurs by converting proportions of 0 and 1 to .5/(N + 1) and 1.5/(N + 1), respectively, where N is the number of trials on which the proportion is based (Hautus, 1995; Miller, 1996).

### Procedure

All three experiments in this article were conducted online, through a video call, using the software Zoom (Zoom Video Communications, Inc., 2020), and stimulus presentation and response recording were controlled with the software E-Prime 3.0 (Psychology Software Tools, Inc., 2016).

Also, in all experiments, informed consent was obtained from participants, and a sociodemographic questionnaire was completed. The main procedure included two phases: study and test phases. Participants were randomly assigned to one of the two conditions: transmission of personal facts or transmission of familiar proverbs. In the condition of familiar proverbs, participants told 50 proverbs to 50 celebrity faces—the 50 proverbs were randomly paired with the 50 faces. They were not told that their memory would be tested later. Each study trial started with a white fixation cross on a black background for 1,000 ms, and then a proverb was presented. After reading silently and memorising the proverb, participants were instructed to press the keyboard spacebar. This resulted in a blank screen for 250 ms, followed by a colour picture of a celebrity face. Here, participants had to say aloud to the face the proverb they had just read and then press the spacebar again, resulting in another blank screen for 250 ms. This procedure was repeated until the participant had told the 50 proverbs to the 50 faces.

For the transmission of personal facts, the procedure was the same. Instead of transmitting familiar proverbs, the participants told 50 personal facts to 50 celebrity faces—the 50 personal facts were randomly paired with the 50 faces. After reading silently, the participant completed and memorised the personal fact and then pressed the keyboard spacebar. This resulted in a blank screen for 250 ms, followed by a colour picture of a celebrity face. Here, participants had to say aloud to the face the complete personal fact and then press the spacebar again, resulting in another blank screen for 250 ms. This procedure was repeated until the participant had told all the 50 personal facts to the 50 faces.

After the study phase, all participants completed two recognition memory tests presented in a counterbalanced order: the item and the destination memory tests. These two tests used entirely nonoverlapping sets of stimuli to prevent cross-test contamination. The item memory test randomly presented 20 faces and 20 facts (20 familiar proverbs or 20 personal facts, depending on the participant’s condition). Half of the items were stimuli previously presented in the study phase, and the other half were not. The participant had to indicate whether that item had appeared during the study phase. The participant responded “yes” by pressing the “c” key on the computer keyboard or “no” by pressing the “m” key. Once a response was made, a blank screen was displayed for 250 ms, and the subsequent test trial followed.

On the destination memory test, 40 face–familiar proverb pairs or 40 face–personal fact pairs, depending on the participant’s condition, were shown in random order: Twenty pairs had been presented during the study phase, and the other 20 were presented with random re-pairings of previously studied facts and faces. Participants reported whether they had once told that fact to that face. “Yes” and “no” responses were made by pressing the same keys as in the item memory test. Each pair remained visible until the participant responded. After each answer, a blank screen was displayed for 250 ms, and the subsequent test trial was presented. The entire procedure took approximately 20–30 min.

## Results

The mean proportions of hits, false alarms, *dʹ*, and C values are shown in Table 1. The software used for the data analysis was JASP 0.11.1 (Jeffreys’s Amazing Statistics Program [JASP] Team, 2021).

**Table 1.** Experiment 1: mean proportion of hits, false alarms, dʹ, and C to item memory and destination memory as a function of condition.

	Hits	False alarms	*dʹ*	C
Personal facts				
Item memory: facts	.97 (.07)	.04 (.07)	3.09 (.43)	.00 (.31)
Item memory: faces	.80 (.21)	.11 (.10)	2.18 (.77)	.13 (.56)
Destination memory	.64 (.15)	.44 (.20)	0.57 (.73)	˗.14 (.44)
Familiar proverbs				
Item memory: facts	.86 (.17)	.03 (.04)	2.73 (.56)	.29 (.43)
Item memory: faces	.85 (.15)	.07 (.08)	2.49 (.56)	.22 (.51)
Destination memory	.69 (.12)	.32 (.15)	1.07 (.59)	˗.01 (.42)

Standard deviation of the mean is reported in parentheses.

To understand whether the Type of Fact and the Type of Test Stimuli influenced item memory, we used a 2 (Type of Fact: familiar proverbs vs. personal facts) × 2 (Type of Test Stimuli: facts vs. faces) mixed Analysis of Variance (ANOVA), performed on the *dʹ* data, with the Type of Fact as the between-participants factor and Type of Test Stimuli as the within-participants factor. There was a significant main effect of the Type of Test Stimuli, *F*(1, 39) = 22.79, *p* < .001, η_p_^2^ = .37, showing that item memory was higher for the facts (*M* = 2.91, *SD* = .50) than for faces (*M* = 2.33, *SD* = .67). Also, there was a significant interaction between the Type of Fact and the Type of Test Stimuli, *F*(1, 39) = 7.63, *p* = .01, η_p_^2^ = .16, indicating a better memory for the facts (*M* = 3.09, *SD* = .43) than the faces (*M* = 2.18, *SD* = .77), only when participants transmitted personal facts. As there was no main effect of Type of Fact (personal facts vs. familiar proverbs), *F*(1, 39) = 0.03, *p* = .86, η_p_^2^ = .0008, we can conclude that the type of information transmitted to the celebrities did not influence the participants’ item memory. In addition, using C criteria, we ran a mixed 2 × 2 ANOVA considering the same variables and observed no significant main effect of the Type of Test Stimuli, *F*(1, 39) = 0.10, *p* = .75, η_p_^2^ = .003, no significant main effect of the Type of Fact, *F*(1, 39) = 2.91, *p* = .10, η_p_^2^ = .07, or interactions, *F*(1, 39) = 1.39, *p* = .25, η_p_^2^ = .03.

To understand whether the Type of Fact could influence destination memory, an independent samples t-test was performed on *dʹ* data. We compared performance when transmitting personal facts versus familiar proverbs. This analysis revealed that destination memory was worse when participants shared personal facts (*M* = .57, *SD* = .73) than when they shared familiar proverbs (*M* = 1.07, *SD* = .59), *t*(39) = 2.40, *p* = .02, Cohen’s *d* = .75, 95 % *CI* [.11, 1.38], replicating the results previously found (Gopie & MacLeod, 2009; T. L. Johnson & Jefferson, 2018). Finally, when applying the t-test applied to the response bias (C), it was not observed a statistically significant difference between the conditions, *t*(39) = 0.93, *p* = .36, Cohen’s *d* = .29, 95 % *CI* [˗.33, 0.91].

### Discussion

The goal of Experiment 1 was to observe whether the poorer destination memory for personal facts would also be observed when participants conveyed familiar proverbs in the control condition. The results support the hypothesis that when self-focus was increased by telling personal facts to celebrity faces, participants’ destination memory performance was worse regardless of whether they conveyed familiar proverbs or interesting facts in the control condition. This is an expected result (i.e., better destination memory for familiar proverbs than personal facts) because it was previously observed that transmitting familiar proverbs leaves more attentional resources to memorise the fact–face association than sharing unfamiliar proverbs (El Haj et al., 2015). Despite personal facts also being familiar information for the participants, this type of fact drives attention to the self (i.e., a higher internal attentional focus), resulting in fewer attentional resources available to associate the information with the destination person (Gopie & MacLeod, 2009; T. L. Johnson & Jefferson, 2018).

Regarding item memory, better memory for the facts than the faces was observed in the personal facts condition. Despite being two different types of material (facts—verbal vs. faces—visual) and comparisons between them should be made with caution, better memory for facts than faces when participants transmitted personal facts can support the idea that when participants convey personal information, the attentional focus is on the information. It was not observed significant differences between the memory for the facts and the faces in the condition of familiar proverbs.

## Experiment 2

In Experiment 1, we observed worse destination memory when participants transmitted personal facts than when they shared familiar proverbs. This result supports the hypothesis that when people’s self-focus is increased by telling personal facts to celebrity faces, their destination memory performance should decrease. This result aligned with the results observed by Jurica and Shimamura (1999) regarding source memory, where remembering the source of information was disrupted if participants were required to answer questions on person-oriented topics. Although source memory decreased when participants generated answers to personal questions, Jurica and Shimamura (1999) observed an increase in item memory. In Experiment 1, we did not observe better item memory for personal facts than familiar proverbs.

A difference between our studies was the type of design applied. Jurica and Shimamura (1999) used a within-participants design. As we wanted to replicate the Gopie and MacLeod (2009) paradigm, in Experiment 1, we applied a between-participants design. Nevertheless, in many studies, a between-participants design has been shown to reduce or eliminate the generation effect (e.g., Hertel, 1989; Schmidt, 1992).

Indeed, most of the studies in the destination memory area have implemented between-participants designs because their goal has been to compare the destination memory performance of a normative population with populations with several disorders (e.g., Alzheimer’s disease: El Haj et al., 2013; Schizophrenia: El Haj et al., 2017; Korsakoff’s syndrome: El Haj, Kessels, et al., 2016; Huntington’s disease: El Haj, Caillaud, et al., 2016) and between young and older samples (El Haj et al., 2013; Gopie et al., 2010). Few studies have applied a within-participants design (Barros et al., 2021; El Haj et al., 2018; El Haj & Ndobo, 2021), and most of these studies carried out manipulations on faces, like the presence of distinctive features on faces (Barros et al., 2021) or the degree of attractiveness of faces (El Haj & Ndobo, 2021). Therefore, in Experiment 2, we applied a within-participants design to observe whether generating and transmitting personal facts harmed destination memory but positively affected item memory, the pattern that occurred for source memory (Jurica & Shimamura, 1999).

## Method

### Participants

The sample consisted of 30 undergraduate students (26 females) with ages ranging between 18 and 27 (*M_age_* = 19.57, *SD* = 2.28). The sample size was calculated *a priori* through G*Power (Faul et al., 2007), targeting a paired samples t-test and using an alpha (α) of .05, a medium effect size (Cohen’s *d* = .50), and a statistical power of .80. As we used a different experimental design (within-participants design), we decided to be more conservative in the effect size. A medium effect size was chosen considering the study by Barros et al. (2021, Exp. 1), where a within-participants design was applied. Participants were native Portuguese speakers and had normal or corrected-to-normal vision. Written consent was obtained from all participants who received course credits for their participation. The local Ethics Committee approved this experiment.

### Materials

Materials were the same as those used in Experiment 1. However, considering that we applied a within-participants design, of the 60 personal facts and the 60 familiar proverbs presented in Experiment 1, 30 personal facts and 30 familiar proverbs were randomly selected for each participant.

### Design

The independent variables were the Type of Fact (personal facts vs. familiar proverbs) and the Type of Test Stimuli (facts vs. faces). Both variables were manipulated through a within-participants design. All participants transmitted personal facts and familiar proverbs and observed facts and faces. To measure our dependent variable, the sensitivity (*dʹ*) score and the response bias (C) score were calculated for both memory tests (item memory and destination memory).

### Procedure

The procedure was the same as Experiment 1, except for the design applied. So, instead of transmitting 50 familiar proverbs or 50 personal facts, participants shared 25 familiar proverbs and 25 personal facts with 50 celebrity faces—the 25 familiar proverbs and 25 personal facts were randomly paired with the 50 faces. As in Experiment 1, they were not informed that their memory would be tested later. This procedure was repeated until the participant had told all 25 familiar proverbs and 25 personal facts to the 50 faces. After the study phase, all participants completed the two recognition memory tests presented in a counterbalanced order: the item and the destination memory tests, as in Experiment 1.

## Results

The mean proportions of hits, false alarms, *d*ʹ, and C values are shown in Table 2.

**Table 2.** Experiment 2: mean proportion of hits, false alarms, dʹ, and C to item memory and destination memory as a function of condition.

	Hits	False alarms	*dʹ*	C
Personal facts				
Item memory: facts	.97 (.08)	.00 (.00)	2.68 (.21)	.07 (.18)
Item memory: faces	.78 (.24)	.17 (.17)	1.70 (.65)	.08 (.57)
Destination memory	.64 (.20)	.51 (.21)	.37 (.78)	˗.25 (.52)
Familiar proverbs				
Item memory: facts	.80 (.17)	.04 (.10)	2.10 (.52)	.33 (.40)
Item memory: faces	.85 (.21)	.19 (.21)	1.83 (.76)	˗.07 (.54)
Destination memory	.64 (.18)	.39 (.20)	0.73 (.76)	˗.02 (.57)

Standard deviation of the mean is reported in parentheses.

To observe whether the Type of Fact and the Type of Test Stimuli influenced item memory, we used a 2 (Type of Fact: familiar proverbs vs. personal facts) × 2 (Type of Test Stimuli: facts vs. faces) repeated measures ANOVA, performed on the *dʹ* data, with the Type of Fact and the Type of Test Stimuli as the within-participants factors. There was a significant main effect of the Type of Test Stimuli, *F*(1, 29) = 29.73, *p* < .001, η_p_^2^ = .51, showing that item memory was higher for facts (*M* = 2.40, *SD* = .40) than for faces (*M* = 1.77, *SD* = .10). Also, the main effect of Type of Fact was significant, *F*(1, 29) = 6.21, *p* = .02, η_p_^2^ = .18, showing better item memory in the condition of personal facts (*M* = 2.19, *SD* = .69) than in the condition of familiar proverbs (*M* = 1.97, *SD* = .19). Finally, there was a significant interaction between the Type of Fact and the Type of Test Stimuli, *F*(1, 29) = 11.93, *p* = .002, η_p_^2^ = .29, showing better memory for the facts (*M* = 2.68, *SD* = .21) than the faces (*M* = 1.70, *SD* = .65), only when participants transmitted personal facts.

In addition, we ran a mixed 2 × 2 repeated measures ANOVA using C criteria considering the same variables. A main effect of the Type of Test Stimuli was observed, *F*(1, 29) = 5.38, *p* = .03, η_p_^2^ = .16, with facts having a more conservative criterion than faces. Also, an interaction was observed between the Type of Fact and the Type of Test Stimuli, *F*(1, 29) = 10.28, *p* = .003, η_p_^2^ = .26, showing a more conservative criterion for the facts than the faces only when participants transmitted familiar proverbs.

To understand whether the Type of Fact influenced destination memory performance, a paired samples t-test was performed on *dʹ* data, in which we compared the transmission of personal facts with the transmission of familiar proverbs. The analysis revealed that destination memory was worse when participants shared personal facts (*M* = 0.37, *SD* = .78) than when they shared familiar proverbs (*M* = .73, *SD* = .76), *t*(29) = 2.16, *p* = .03, Cohen’s *d* = .40, 95 % *CI* [.02, .76],^4^ replicating the results previously found (Gopie & MacLeod, 2009; T. L. Johnson & Jefferson, 2018). Finally, when applying the t-test applied to the response bias (C), it was not observed a statistically significant difference between the conditions, *t*(29) = 1.95, *p* = .06, Cohen’s *d* = .36, 95 % *CI* [˗.02, .72].

### Discussion

Experiment 2 was designed to observe whether generating and transmitting personal facts harmed destination memory but positively affected item memory, as has been reported for source memory (Jurica & Shimamura, 1999). For this reason, we decided to examine the same independent variables but applying a within-participants design, which would complement the previous studies because all the previous studies that observed the effect of the transmission of personal facts on destination memory used a between-participants design (Gopie & MacLeod, 2009; T. L. Johnson & Jefferson, 2018).

In this experiment, similar to the results observed in Experiment 1 (between-participants design), destination memory was worse when participants transmitted personal facts than when they shared familiar proverbs. Also, similar to Experiment 1, better memory for facts than faces was observed only when participants transmitted personal facts, supporting the idea that participants’ attentional focus was on the information when personal facts were shared. Again, significant differences between memory for facts and faces were not observed when transmitting familiar proverbs.

More interestingly, as noted in source memory studies (Jurica & Shimamura, 1999), we observed better memory for personal facts than familiar proverbs, which the generation effect can explain because self-generated material is better remembered than information that is only read (see Bertsch et al., 2007, for a review).

## Experiment 3

An enhanced generation effect for item memory and an impaired generation effect for source memory were observed in previous studies (Jurica & Shimamura, 1999). This result was also observed in Experiment 2 regarding destination memory. However, it is essential to note that this pattern of results (i.e., positive generation effect for item memory and negative generation effect for source memory) occurred when both personal and non-personal information were presented (Jurica & Shimamura, 1999). The participants generated answers to generic preferences (Experiment 2—e.g., “What type of sports are commonly watched on television?”) and general knowledge (Experiment 3—e.g., “What is the name of the comic strip character who eats spinach to increase his strength?”) presented by one of three faces. Just as it happened for personal questions, remembering the source of information was disrupted when participants were required to answer questions on non-person topics instead of reading statements. Jurica and Shimamura (1999) assumed that biases in the use of personal questions could not account for finding a positive generation effect for item memory and a negative generation for source memory. They proposed that the demands involved in a generation task cause participants to pay attention to the item, which benefits the encoding of item-specific information but at the cost of encoding associations between the item and other environmental elements (Jurica & Shimamura, 1999).

So, the question is whether the worse destination memory observed when participants had to generate and transmit personal facts compared with when they only conveyed familiar proverbs can be attributed to the facts being personal and generated. With this in mind, our experiment aimed to observe whether the generation of familiar proverbs decreased destination memory (presumably due to a shift in the attentional focus).

As the information transmitted is self-generated, we hypothesised that fewer attentional resources are available to associate the generated familiar proverbs with the destination face. So, we hypothesised that the generation of familiar proverbs would lead to worse destination memory performance than the transmission (without generation) of familiar proverbs, which shows a negative generation effect in destination memory.

## Method

### Participants

The sample consisted of 31 undergraduate students (15 females) with ages ranging between 18 and 23 (*M_age_* = 19.26, *SD* = 1.41). The sample size was calculated *a priori* through G*Power (Faul et al., 2007), targeting a paired samples t-test and using an alpha (α) of .05, a medium effect size (Cohen’s *d* = .50), and a statistical power of .80. A medium effect size was chosen considering Experiment 2 (Cohen’s d = .40). Participants were native Portuguese speakers and had normal or corrected-to-normal vision. Written consent was obtained from all participants who received course credits for their participation. The local Ethics Committee approved this experiment.

### Materials

The materials were the same as those in Experiments 1 and 2. From the 60 familiar proverbs, two lists of 30 proverbs each were created, with similar values for length (*M_List1_* = 6.37; *M_List2_* = 6.50), familiarity (*M_List1_* = 4.64; *M_List2_* = 4.67), and valence (*M_List1_* = 3.13; *M_List2_* = 3.12). It is important to note that the stimuli were counterbalanced so that each proverb was presented complete for half of the participants (e.g., “Out of sight, out of mind”) and incomplete for the rest of the participants (e.g., “Out of sight, out of . . .”).

### Design

The independent variables were the Type of Processing (generation vs. transmission) of familiar proverbs and the Type of Test Stimuli (facts vs. faces). They were manipulated in a within-participants design. All participants transmitted familiar proverbs and generated familiar proverbs, and all participants observed facts and faces. Again, to measure our dependent variable, the sensitivity (*dʹ*) score and the response bias (C) score were calculated for both memory tests (item memory and destination memory).

### Procedure

The procedure was the same as Experiment 2. However, instead of transmitting 25 familiar proverbs and 25 personal facts, participants transmitted 50 familiar proverbs randomly associated with 50 celebrity faces. Half of the proverbs were presented complete (e.g., “Out of sight, out of mind”), and the other half needed to be completed (e.g., “Out of sight, out of . . .”) with one word. Participants were not told that their memory would be tested later. This procedure was repeated until the participant had told 25 complete familiar proverbs and completed and told 25 incomplete familiar proverbs to the 50 faces. After the study phase, all participants completed two recognition memory tests presented in a counterbalanced order: the item and the destination memory tests, as in Experiments 1 and 2.

## Results

The mean proportions of hits, false alarms, *d*ʹ, and C values are shown in Table 3.

**Table 3.** Experiment 3: mean proportion of hits, false alarms, dʹ, and C to item memory and destination memory as a function of condition.

	Hits	False alarms	*dʹ*	C
Generation of familiar proverbs				
Item memory: facts	.91 (.10)	.04 (.10)	2.42 (.42)	.12 (.30)
Item memory: faces	.77 (.21)	.12 (.18)	1.81 (.71)	.18 (.50)
Destination memory	.67 (.21)	.42 (.21)	0.74 (.87)	˗0.15 (.57)
Transmission of familiar proverbs				
Item memory: facts	.77 (.23)	.02 (.08)	2.07 (.63)	.38 (.43)
Item memory: faces	.81 (.23)	.12 (.18)	1.89 (.89)	.14 (.45)
Destination memory	.64 (.23)	.39 (.18)	0.74 (.92)	˗0.03 (.51)

Standard deviation of the mean is reported in parentheses.

To understand whether the Type of Processing and the Type of Test Stimuli influenced item memory, we used a 2 (Type of Processing: generation vs. transmission) × 2 (Type of Test Stimuli: facts vs. faces) repeated measures ANOVA, performed on the *dʹ* data, with both variables as within-participants factors. There was a significant main effect of the Type of Test Stimuli, *F*(1, 30) = 8.89, *p* = .01, η_p_^2^ = .23, showing that item memory was higher for facts (*M* = 2.24, *SD* = .56) than for faces (*M* = 1.85, *SD* = .80). Also, there was a marginally significant interaction between the Type of Processing and the Type of Test Stimuli, *F*(1, 30) = 4.07, *p* = .05, η_p_^2^ = .12. Post hoc tests showed better memory for the facts (*M* = 2.42, *SD* = .42) than the faces (*M* = 1.81, *SD* = .71), only when participants generated familiar proverbs (*p* = .004). As there was no main effect of Type of Processing (generation vs. transmission), *F*(1, 30) = 1.19, *p* = .28, η_p_^2^ = .04, we can conclude that generating or transmitting familiar proverbs to the celebrities did not influence the participants’ item memory.

In addition, using C criteria, we ran a mixed 2 ×2 ANOVA considering the same variables. There was a significant interaction between the Type of Processing and the Type of Test Stimuli, *F*(1, 30) = 6.28, *p* = .02, η_p_^2^ = .17, showing a more conservative criterion for the facts in the condition of transmission of familiar proverbs (*M* = .38, *SD* = .43) than in the condition of the generation of familiar proverbs (*M* = .12, *SD* = .30). No significant main effect of the Type of Processing, *F*(1, 30) = 2.63, *p* = .12, η_p_^2^ = .08 and no significant main effect of the Type of Test Stimuli, *F*(1, 30) = 1.11, *p* = .30, η_p_^2^ = .04, was observed.

To observe whether the Type of Processing influenced destination memory, a paired samples t-test was performed on *dʹ* data, in which we compared the transmission of familiar proverbs with the generation of familiar proverbs. Significant differences were not observed between the two conditions, *t*(30) = .03, *p* = .98, Cohen’s *d* = .005, 95 % *CI* [˗.35, .36]. In other words, generating familiar proverbs (*M* = .74, *SD* = .87) did not lead to worse destination memory than did transmitting familiar proverbs (*M* = .74, *SD* = .92). Finally, when applying the t-test applied to the response bias (C), a statistically significant difference between the conditions was not observed, *t*(30) = 1.07, *p* = .29, Cohen’s *d* = .19, 95 % *CI* [˗.16, .55].

Despite being familiar proverbs, not all proverbs were known to the participants. In this case, participants were instructed to complete the proverbs with any that came to mind. However, it was not observed significant differences in the destination memory task between the proverbs that they completed correctly and those that they completed with anything that came to their mind (Recognition _filled correctly_ = .22; Recognition _filled incorrectly_ = .19), *t*(25) = .41, *p* = .69, Cohen’s *d* = .08, 95 % *CI* [˗.46, .31].

### Discussion

In this experiment, we aimed to observe whether the generation of familiar proverbs influenced the destination memory. However, significant differences between the two conditions were not observed, which means that generating familiar proverbs does not lead to worse destination memory than only transmitting. With these results, we can assume that the poorer destination memory observed when participants generated personal facts is not the result of participants generating information. If information generation could explain this poorer destination memory, worse performance would have been observed when participants generated familiar proverbs.

It is important to note that, despite being familiar proverbs, not all proverbs were known to the participants. In this case, participants were instructed to complete the proverbs with any completion that came to mind. However, material generated freely could be somehow different and thus more memorable (McCurdy et al., 2017), leading to a context memory consistently better. For this reason, we compare destination memory performance when participants knew the proverbs and completed them correctly with when they did not know the proverbs and completed them with whatever came to mind and were not observed significant differences.

Also, it is interesting to note that better memory for the facts than faces was observed when participants generated familiar proverbs. This result was observed in Experiments 1 and 2 when participants generated and transmitted personal facts, supporting the idea that participants’ attentional focus is on the information when information is generated. It was not observed significant differences between memory for the facts and the faces when transmitting familiar proverbs.

Regarding the criterion response, there was a difference in the item memory task between the complete familiar proverbs (i.e., facts presented in the condition of transmitting familiar proverbs) and the incomplete familiar proverbs (i.e., facts presented in the condition of the generation of familiar proverbs). A difference in criterion response was also observed in Experiment 2 between personal facts and familiar proverbs. Participants adopted a more conservative criterion in the transmission of familiar proverbs compared with the condition of the generation of familiar proverbs and the condition of the generation of personal facts. It seems that a conservative criterion shift occurred, where participants adopted a more liberal response criterion for the facts in the condition of generating information (personal and no personal).

## General Discussion

Destination memory studies that involve participants transmitting personal facts (e.g., *my favourite colour is* . . .) always make a comparison with interesting facts (e.g., *a shrimp’s heart is in its head*) (Gopie & MacLeod, 2009; T. L. Johnson & Jefferson, 2018), where personal facts were generated and transmitted, and interesting facts were only transmitted. So, with this set of experiments, we wanted to disentangle the impact of the generation effect on destination memory and item memory using another type of stimuli and experimental design.

Thus, the goal of Experiment 1 was to replicate worse destination memory when personal facts were transmitted but using different material: instead of interesting facts, we presented familiar proverbs in the control condition. In addition to being a type of stimulus widely used in the destination memory area, choosing familiar proverbs allowed us to present familiar information in both conditions. In addition, all of the previous studies that observed the influence of the transmission of personal facts on destination memory applied a between-participants design (Gopie & MacLeod, 2009; T. L. Johnson & Jefferson, 2018). To observe a possible influence of the generation effect on item and destination memory, in Experiment 2, we decided to explore the same independent variables—transmission of personal facts versus familiar proverbs—but applying a within-participants design.

As hypothesised, worse destination memory was observed when participants transmitted personal facts than familiar proverbs no matter which design was implemented: between-participants design (Experiment 1) or within-participants design (Experiment 2). This was an expected result (i.e., better destination memory for familiar proverbs than personal facts) because even though both sets of facts (proverbs and personal) were familiar information for the participants, the higher internal attentional focus driven by the transmission of personal facts resulted in less attentional resources and, consequently, worse destination memory.

Experiment 3 allowed us to reject the explanation that worse memory is observed due to the generation effect. If the information generation could explain the worse destination memory, worse destination memory would have also been observed when participants generated familiar proverbs. Despite Jurica and Shimamura (1999) observing a negative generation effect for source memory, presenting personal and non-personal information, our results did not show a negative generation effect for destination memory with non-personal information.

Like source memory, destination memory is autobiographical because it is recollected in the context of a specific time and place concerning oneself as a participant in the episode. However, source and destination memory differ regarding the direction of information transfer—output in the case of destination memory and input in the case of source memory. According to Gopie and MacLeod (2009), when a person receives information from someone else, attention is directed to the incoming information and to the person who is providing it, and the attention to both the information and the person will benefit the association between them, leading to good source memory. However, in Jurica and Shimamura’s (1999) study, when participants were asked to generate answers to personal and non-personal questions presented by one of three faces, the higher attentional focus was on the information/answer that had to be generated and not on the question or the person who was asking it, which resulted in worse source memory.

Generating information may influence destination memory less than source memory because the attentional focus when transmitting information to someone else is already on the information (Gopie & MacLeod, 2009), both when sharing and generating information. However, when participants were asked to generate and transmit personal facts, this promoted a higher internal attentional focus (i.e., higher self-focus), where the attentional focus is on themselves and on the information, which leaves fewer attentional resources to associate the outgoing information with the person that one is telling it to. Thus, although generating non-personal information does not result in worse destination memory (Experiment 3), generating personal information does lead to worse destination memory (Experiment 2).

Regarding item memory, we did not replicate a generation effect for item memory in Experiment 1 (using a between-participants design) and in Experiment 3 (where participants generated familiar proverbs). Indeed, better item memory for personal facts than familiar proverbs was only observed in Experiment 2. One possible explanation is the type of design applied because, in many studies, a between-participants design has been shown to reduce or even eliminate the generation effect (e.g., Hertel, 1989; Schmidt, 1992), and for this reason, a generation effect for item memory was not observed in Experiment 1. However, this result was not observed in Experiment 3, where participants generated familiar proverbs compared with the transmission (without generation) for familiar proverbs in a within-participants design.

These different results concerning the generation effect on item memory may be related to one limitation of this study, which also occurred in the study of Jurica and Shimamura (1999): Participants were not tested on the items they had generated. In the study of Jurica and Shimamura, participants were asked to identify who asked the question presented (e.g., who asked, “What is the name of the comic strip character who eats spinach to increase his strength?”) and had not been presented at any time with the generated information (e.g., Popeye). In our experiments, participants were asked to identify to whom they transmitted the personal facts (e.g., “my favourite colour is . . .) or the proverbs (e.g., “Haste is the enemy of . . .”), not being exposed to the information generated. So, in future studies, to have a better understanding of the generation effect on item memory, participants must be exposed to the generated material (e.g., proverbs and personal facts). Although we do not know how they will be generated in advance, the researcher can record how the participants completed the personal facts, for example. Later, in the test phase, we could present the answers the participants generated to complete the personal facts instead of giving incomplete personal facts.

It is also interesting to note that a difference between facts and faces was consistently observed in the condition where the participant had to generate information (personal facts or familiar proverbs), which could be taken as support for the idea that when participants generate information, the attentional focus is on the information. However, better item memory for the facts than the faces does not mean poorer destination memory will necessarily occur. Worse destination memory was observed only when the generated information was personal.

In sum, in these three experiments, worse destination memory when participants transmitted personal facts was observed even when another type of fact was presented in the control condition, namely familiar proverbs, and in another design, a within-participants design. Also, these experiments allowed us to reject the generation effect as an explanation for the worse destination memory when personal facts were transmitted. These results thus seem to support previous results (Gopie & MacLeod, 2009; T. L. Johnson & Jefferson, 2018) that claimed that sharing personal facts promotes a higher internal attentional focus, resulting in less attentional recourses and worse destination memory.

## Notes

1. An approximate English translation of the Portuguese proverb is “Haste is the enemy of perfection.”

2. An approximate English translation of the personal fact is “My favorite color is . . .”

3. Negative dʹ were not excluded because this is not the usual data analysis procedure in the destination memory area. This type of data is frequent and reflects the difficulty of the associative memory task.

4. In Experiment 2, some participants obtained negative dʹ, and when these participants were eliminated, a loss in the effect size occurred (a Cohen’s d = .18 instead of a Cohen’s d = .40), slightly changing the results. However, as eliminating negative dʹ is not the usual data analysis procedure in the destination memory area, we opted to maintain this data.
